# Preanalytical Errors in Hematology: Insights From a Tertiary Care Hospital

**DOI:** 10.7759/cureus.69641

**Published:** 2024-09-18

**Authors:** Vallal Kani, Kavitha Kannan, Sumithra Arumugam, Sulochana Sonti

**Affiliations:** 1 Department of Pathology, Saveetha Medical College and Hospitals, Saveetha Institute of Medical and Technical Sciences, Saveetha University, Chennai, IND

**Keywords:** haematology, hematology, pre-analytical errors, quality control, sample rejection, turnaround time

## Abstract

Introduction

Errors occur in the laboratory at any level of the testing process. Recognizing these errors may cause patient distress by delaying diagnosis and management until results are released. This study aims to assess preanalytical errors in the laboratory and suggests methods to prevent them to improve accuracy and efficiency in the hematology department of a tertiary care hospital. The background emphasizes the critical role of preanalytical procedures in ensuring accurate hematological diagnoses and highlights the frequent mistakes that occur during specimen collection, handling, and transportation. Staff training, process standardization, using suitable collecting and transport equipment, putting quality control measures in place, and using automation technologies are some strategies for reducing errors and speeding up turnaround times. To increase diagnostic accuracy, patient care outcomes, and laboratory efficiency, hematology laboratories should address preanalytical mistakes and employ fostering methods.

Aims and objectives

The objective of this research is to evaluate the frequency and characteristics of preanalytical errors within the hematology laboratory of a tertiary care hospital. The study seeks to gauge the scope of the problem, identify key factors contributing to these errors, such as issues in specimen collection, handling, and transportation, and highlight areas where improvements can be made. Also, it intends to assess how preanalytical errors affect hematological accuracy and turnaround times, with a focus on patient care outcomes, and recommend techniques to reduce preanalytical errors and improve the accuracy of hematological results.

Materials and methods

This study was conducted at the hematology laboratory of our hospital from January 2023 to June 2024 after getting proper approval from the Institutional Review Board (IRB approval number 294/08/2024/PG/SRB/SMCH). It is a retrospective analytical study, and the study population comprised samples of patients from the emergency, inpatient, and outpatient departments, which included 51,155 complete blood count (CBC) and 5,449 peripheral smear (PS) samples. The study included only test samples sent specifically for hematological analysis, while those sent for cytological, biochemical, or microbiological testing were excluded. The distribution frequency of the samples and the number of rejected samples were analyzed, and the results were then compared and correlated.

Results

During the study period, a total of 56,604 samples were processed in the hematology laboratory. Of these, 1018 samples (1.8%) were rejected due to preanalytical errors. The most frequent error was submitting an insufficient sample (52.3%), while the least frequent was the use of an empty or defective tube (0.3%). In the emergency department, the primary issues were insufficient and clotted samples, while in pediatric cases, errors were mainly due to inadequate or diluted samples.

Conclusion

Preanalytical errors in hematology laboratories, though often overlooked, can significantly impact diagnostic accuracy and patient care. Our study highlights that a substantial portion of errors arise from inadequate sample collection and handling, particularly in emergency and pediatric cases. Adherence to standard laboratory techniques can dramatically reduce preanalytical errors.

## Introduction

Accurate laboratory test results are crucial for modern diagnoses; hence, these results must be reliable and accurate [1]. A medical lab provides rapid and precise results for laboratory tests, which are vital for patient care [2]. The hematological laboratory is a high-volume sector in clinical laboratories. Hematology tests are commonly accessible, even in small laboratories with limited services [1]. Quality assurance in hematology labs guarantees accurate and dependable test results for users [3]. The goal of quality assurance in the lab is a consistent, trustworthy test result [4]. Providing safe healthcare requires high-quality medical diagnostics [5].

A laboratory's total testing procedure (TTP) includes all steps from requisitioning tests to obtaining the results [6]. Accurate and reliable laboratory test results are essential for clinical decision-making, as they influence approximately 70 percent of cases [1]. Laboratory test results have a crucial role in clinical decisions, including admission, prescription, and discharge [7]. Mistakes in clinical laboratories can lead to increased healthcare costs and reduced patient satisfaction [8]. These errors occur at various stages of the testing process and may stem from miscommunication, actions by individuals, or poorly designed procedures [9].

Laboratory errors can delay diagnosis and treatment, causing patient inconvenience and anxiety. In some cases, specimens cannot be retaken, resulting in missed opportunities for diagnosis or screening. Errors that go undetected before data are released can lead to incorrect or missing diagnoses, unnecessary testing or treatment, and put patient safety at risk [10]. Errors in laboratory sample processing fall into three categories: preanalytical, analytical, and post-analytical [4]. Statland and Winkel coined the term preanalytical phase in 1977 [11]. Although automation has minimized errors in the analytical and post-analytical phases, the preanalytical stage remains largely dependent on manual work [12].

Despite the constant development of automation, human interaction can still impact laboratory results, making them preventable [1]. To ensure accurate reporting of results, all three stages of the testing process must be free of errors. Preanalytical errors during laboratory procedures have been associated with heightened risks to patient safety [13]. The preanalytical phase starts with the clinical order for a laboratory test and extends through the sample preparation for analysis. Studies indicate that only 7-13% of errors occur during the analytical phase; the majority of errors (46-68%) arise during the preanalytical phase, with additional errors (18-47%) continuing into the post-analytical phase [1,9].

Preanalytical errors can happen before or after the sample is received by the laboratory and happen before the analytical phase of the TTP. Research indicates that they contribute significantly to laboratory errors. In addition to reducing the clinical and economic effectiveness of laboratory services, preanalytical errors raise the risk of incorrect or unsuitable treatment interventions, unnecessary follow-up tests, and delayed diagnoses [13]. These errors encompass issues such as improper test ordering, patient misidentification, errors in preparation, sample collection problems, compromised sample quality (e.g., dilution, clotting, hemolysis), the use of incorrect containers and anticoagulants, and problems with sample transportation and storage. The preanalytical step also includes sample sorting, centrifugation, labeling, and separation [10,12]. The objective of this research is to evaluate the frequency and types of preanalytical errors in the hematology laboratory and to propose methods for minimizing these errors, thereby enhancing the accuracy of hematological results.

## Materials and methods

The study was carried out at the Hematology Laboratory of Saveetha Medical College and Hospitals, Saveetha Institute of Medical and Technical Sciences, Saveetha University, Chennai, between January 2023 and June 2024, following approval from the Institutional Review Board (IRB approval number 294/08/2024/PG/SRB/SMCH). This is a retrospective analytical study using test samples from patients across the emergency, outpatient (OP), and inpatient (IP) departments. The study population included patients undergoing hematological examination and healthcare professionals involved in the preanalytical phase of hematological specimen handling, including clinicians, nurses, laboratory technicians, pathologists, and the staff members responsible for sample collection, transportation, processing, and storage within the hematology laboratory.

During the study period, a total of 56,604 samples were analyzed in the hematology laboratory. This included 51,155 complete blood count (CBC) samples and 5,449 peripheral smear (PS) samples. We retrieved all essential data from the laboratory records, which comprised sample collection and rejection data, and tabulated them in the Microsoft Office Excel 2016 (Microsoft Corporation, Redmond, Washington, United States). The inclusion criteria were the test samples received at hematology and clinical pathology laboratories. The exclusion criteria were the samples sent to cytology, biochemistry, and microbiology laboratories.

The relevant history, clinical details, and investigation details regarding any case were taken from the test request forms and Medical Information Archiving Software (MIAS) database of our hospital. The samples for complete blood count were analyzed using an automated analyzer (Sysmex XN 1000 six-part analyzer). The statistical analysis was conducted and is presented in the form of frequency tables and correlated. The study identified several preanalytical variables that can affect test results. These include the use of incorrect collection tubes, clotted blood, insufficient sample volume, hemolyzed samples, diluted samples, excess sample volume, empty or damaged vacutainers, incorrect labeling, and delays in sample transfer to the laboratory.

## Results

**Figure 1:**
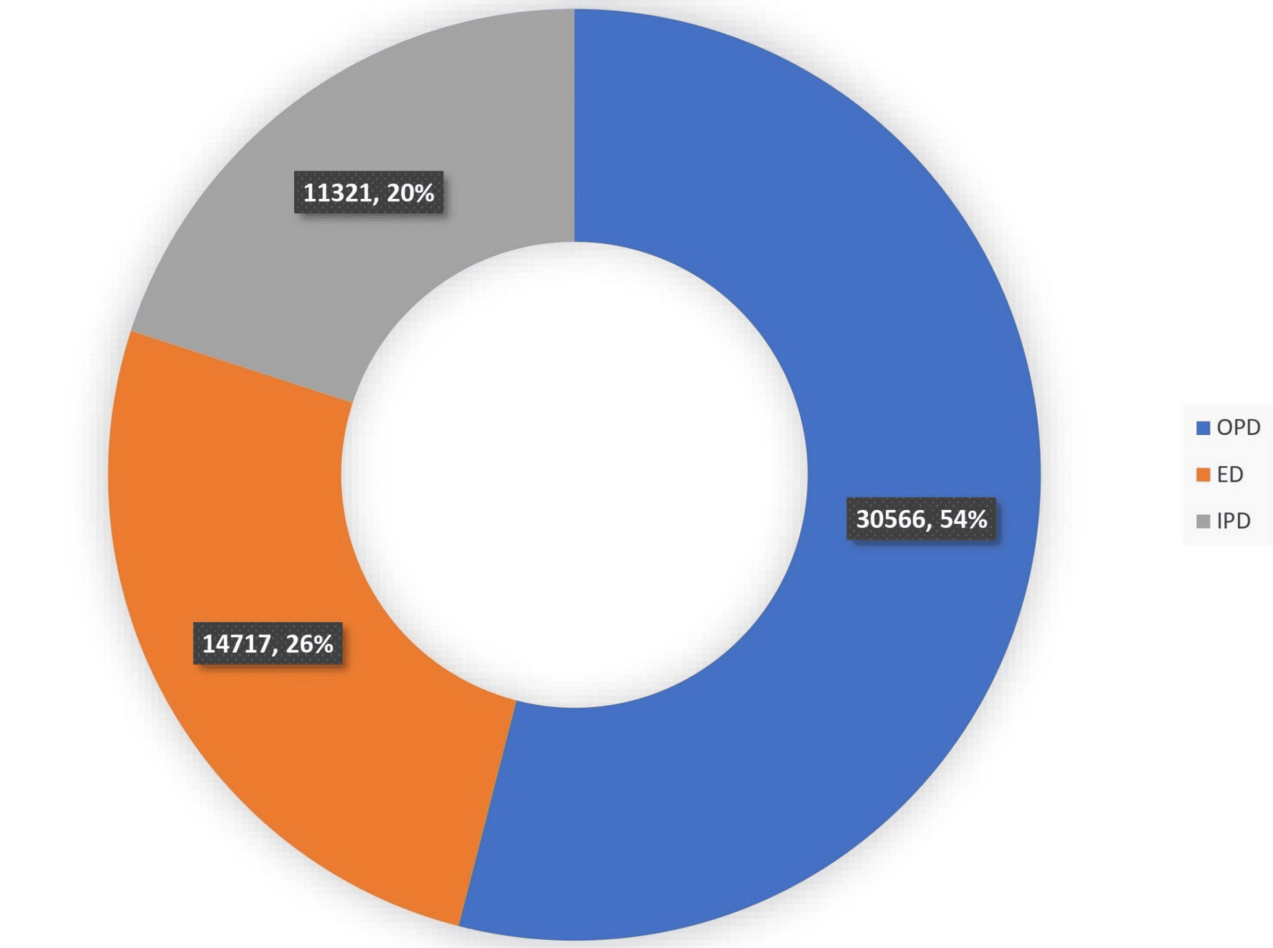
Department-wise distribution of samples received at the hematology laboratory OPD: outpatient department; IPD: inpatient department; ED: emergency department

A total of 56,604 samples received in the hematology laboratory were analyzed during the study period, including 51,155 CBC samples and 5,449 PS samples. Out of these, 30,566 (54%) samples were from the outpatient department (OPD), 14,717 (26%) samples were from the emergency department (ED), and 11,321 (20%) samples were from the inpatient department (IPD), as shown in Figure 1.

Various preanalytical errors led to the rejection of 1,018 (1.8%) samples. In this study, the insufficient sample was the most common preanalytical variable (n=533, 52.3%), and empty/damaged tubes had the least reported error (n=3, 0.3%). A substantial portion of samples was rejected due to clotting, accounting for 21.4% (n=219) (Table 1).

**Table 1:** Distribution of samples rejected due to preanalytical errors

Serial number	Preanalytical errors	Number of samples	Percentage (%)
1	Incorrect test tube	144	14.1%
2	Clot in the sample	219	21.4%
3	Inadequate sample	533	52.3%
4	Hemolzsed sample	65	6.4%
5	Diluted sample	17	1.7%
6	Excessive sample	7	0.7%
7	Error in labeling	12	1.2%
8	Delay in transit	18	1.8%
9	Empty/damaged tubes	3	0.3%
	Total	1018	100%

**Figure 2:**
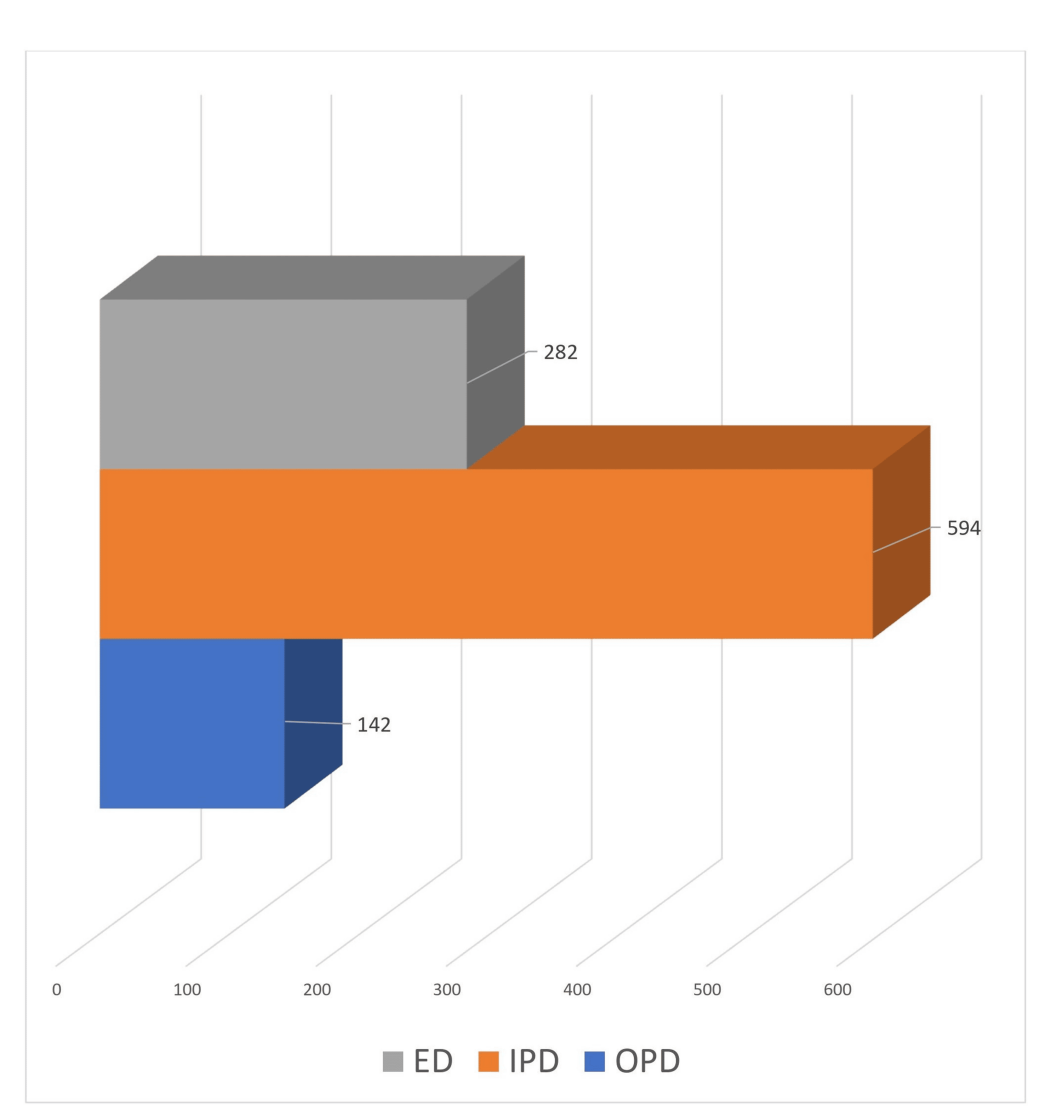
Department-wise distribution of preanalytical errors ED: emergency department; IPD: inpatient department; OPD: outpatient department

Out of the 1,018 preanalytical errors, most of them were from the IPD (n=594, 58.4%), followed by the ED (n=282, 27.7%), and OPD (n=142, 13.9%), as shown in Figure 2.

Table 2 shows the distribution of preanalytical errors among various inpatient departments. It was noted that pediatric samples had the highest preanalytical errors (n=476, 46.8%), followed by emergency department samples (n=335, 32.9%), and the major reasons were the sample insufficiency and clotted sample.

**Table 2:** Distribution of preanalytical errors among various inpatient departments

Serial number	Preanalytical errors	Inpatient departments				
		Medicine	Surgery	Pediatrics	Emergency	Total
1	Inadequate sample	28	58	383	64	533
2	Clot in the sample	10	31	45	133	219
3	Incorrect test tube	18	13	16	97	144
4	Hemolyzed sample	15	12	13	25	65
5	Delay in transit	3	4	8	3	18
6	Diluted sample	3	3	9	2	17
7	Excessive sample	2	2	1	2	7
8	Error in labeling	1	2	1	8	12
9	Empty/damaged tubes	1	1	0	1	3
	Total	81	126	476	335	1018

## Discussion

A laboratory error encompasses any mistake that occurs throughout the testing process, from the initial order to the final report, and negatively impacts the quality of laboratory services [14]. Pre-testing errors account for up to 75% of laboratory errors [14,15]. Categorizing laboratory errors based on severity can help identify those needing immediate attention for quality improvement and adopt preventative actions. Laboratory errors can be minimized by classifying them according to their severity, deploying corrective and preventative measures, and identifying which errors require immediate attention for quality improvement [16]. Poor patient satisfaction is a direct consequence of laboratory errors, which are also linked to financial restrictions [6]. In this retrospective research, we investigated the prevalence and types of preanalytical variables leading to sample rejection. Across various departments in our hospital, a total of 1,018 preanalytical variables were identified, representing 1.8% of the samples, and the findings were concordant with most of the studies conducted at different centers (Table 3).

**Table 3:** Comparison of our results with other studies

Study (Year)	Total number of samples	Total errors (%)	Most common error	Concordance/Discordance
Upreti et al. (2013) [17]	135808	1339 (1%)	Wrong label, insufficient sample	Concordant
Narang V et al. (2014) [3]	471006	1802 (0.38%)	Clotted, insufficient sample	Concordant
Arul P et al. (2018) [7]	118732	513 (0.43%)	Insufficient sample, clotted sample	Concordant
Gaur K et al. (2020) [15]	189104	4052 (2.14%)	Insufficient sample, clotted sample	Concordant
Iqbal MS et al. (2023) [1]	67892	886 (1.30%)	Insufficient sample, clotted sample	Concordant
Present study	56604	1018 (1.80%)	Insufficient sample, clotted sample	Concordant

The prevalence rate in our study was within the expected range for preanalytical errors in hematology, reinforcing that despite technological advances, human factors still play a significant role in laboratory errors. The predominance of insufficient samples (52.3%) and clotted samples (21.4%) in our study mirrors trends reported in other studies, underscoring a widespread issue in hematology laboratories. Insufficient samples can occur due to various factors, including inadequate blood draw technique, difficult venipuncture in patients with challenging veins (such as infants, elderly, or patients with chronic illness), improper handling during sample transportation, and also the fact that capillary blood sample collection increases preanalytical errors [18]. Clotted samples often result from delayed mixing with anticoagulants or improper handling post-collection. The fact that these errors were most prevalent in emergency departments and pediatrics is significant. These areas often deal with patients who may be more difficult to draw blood from, such as uncooperative children or critically ill patients, which can contribute to a higher likelihood of insufficient or clotted samples. Additionally, using intravenous catheters for sample collection can lead to diluted samples. The elevated rate of preanalytical errors in pediatric care underscores the complexity and high likelihood of errors associated with blood sampling in this population [19].

The stress of these high-pressure environments may also lead to more frequent deviations from standard protocols, further exacerbating the issue. Preanalytical errors, particularly those involving insufficient or clotted samples, have a direct impact on the reliability of hematological test results. Insufficient samples may necessitate recollection, leading to delays in diagnosis and treatment, which can be particularly critical in emergency settings. Clotted samples, on the other hand, can lead to inaccurate results, such as falsely elevated platelet counts or other spurious hematological findings, which might mislead clinical decision-making. Moreover, the need for repeat sampling not only delays the diagnosis but also increases the workload on the laboratory staff, potentially leading to further errors. This cyclical effect underscores the importance of minimizing preanalytical errors to maintain the integrity of hematological testing and ensure timely and accurate patient care. Given the findings, it is evident that targeted interventions are necessary to reduce preanalytical errors, especially in high-risk areas like emergency departments and pediatrics. When errors are detected, they often lead to sample rejection at the laboratory before processing. This necessitates resampling, which increases the workload for hospital staff, wastes hospital resources, exacerbates patient discomfort, and delays the reporting of results [20,21,22].

Therefore, implementing certain strategies could minimize these errors. Some specific strategies include enhanced training programs by regular training and retraining of staff on the correct techniques for blood collection and handling. Simulation-based training can be particularly effective in helping staff practice and perfect their skills in a controlled environment. Standardization of protocols by developing and strictly enforcing standardized protocols for sample collection, labeling, transportation, and processing can minimize variations that lead to errors. This could include the use of checklists to ensure all steps are followed correctly. Automation and technological solutions by implementing automated systems for sample handling, such as automated mixing devices for blood samples immediately post-collection, which can reduce the likelihood of clotting.

Additionally, real-time monitoring systems that alert staff to potential errors (e.g., insufficient volume, incorrect labeling) before the sample reaches the laboratory can preempt issues. Focusing on high-risk areas using tailored interventions in high-risk departments is essential. For example, in pediatric units, using age-appropriate collection devices and techniques can help reduce the incidence of insufficient samples. In emergency departments, where time is critical, protocols should be streamlined to ensure rapid yet accurate sample collection and handling.

## Conclusions

Preanalytical errors remain a significant issue in labs due to factors beyond the direct control of the laboratories. Although these errors cannot be eliminated, their occurrence can be significantly reduced. Our study not only highlighted the persistent issue of preanalytical errors in hematology but also provided valuable insights into specific areas where improvements are needed. By addressing these errors through targeted strategies, laboratories can enhance the quality of hematological testing, ultimately leading to better patient outcomes. The alignment of our findings with previous studies reinforces the universal nature of these challenges and underscores the need for widespread adoption of best practices to mitigate them. This more detailed discussion provides a thorough analysis of our study's findings in the context of existing literature and offers actionable recommendations for reducing preanalytical errors.
