# Correction: Preanalytical Errors in Hematology: Insights From a Tertiary Care Hospital

**DOI:** 10.7759/cureus.c342

**Published:** 2025-10-02

**Authors:** Vallal Kani, Kavitha Kannan, Sumithra Arumugam, Sulochana Sonti

**Affiliations:** 1 Department of Pathology, Saveetha Medical College and Hospitals, Saveetha Institute of Medical and Technical Sciences, Saveetha University, Chennai, IND

This article has been corrected to revise the abstract due to unintentional text duplication of Iqbal MS, Tabassum A, Arbaeen AF, Qasem AH, Elshemi AG, Almasmoum H. Preanalytical Errors in a Hematology Laboratory: An Experience from a Tertiary Care Center. *Diagnostics*. 2023; 13(4):591. https://doi.org/10.3390/diagnostics13040591.

Upon close review, it was determined that the text duplication was due to human error on the part of the authors and was not intentional plagiarism. As a result, this correction has been issued.

